# The Dynamic and Fragile Nature of Eyewitness Memory Formation: Considering Stress and Attention

**DOI:** 10.3389/fpsyg.2021.666724

**Published:** 2021-04-13

**Authors:** Alia N. Wulff, Ayanna K. Thomas

**Affiliations:** Cognitive Aging and Memory Laboratory, Department of Psychology, Tufts University, Medford, MA, United States

**Keywords:** inattentional blindness, stress, eyewitness memory, emotional arousal, memory distortions

## Abstract

Eyewitnesses are often susceptible to recollection failures and memory distortions. These failures and distortions are influenced by several factors. The present review will discuss two such important factors, attention failures and stress. We argue that acute stress, often experienced by eyewitnesses and victims of crimes, directly influences attentional processes, which likely has downstream consequences for memory. Attentional failures may result in individuals missing something unusual or important in a complex visual field. Amongst eyewitnesses, this can lead to individuals missing details, even unusual or important central details, regarding the crime. Surprisingly, few studies have investigated attentional failures in eyewitness scenarios, and none have investigated the relationship between stress, attention, and witness memory. This review will discuss the impact of attentional failures, mainly those resulting from inattentional blindness, in applied contexts in order to bridge to eyewitness scenarios. In addition, we will integrate the applied literature on attentional failures with literature that examines the influences of arousal and stress on attention. We will conclude by presenting how future research may tease apart the independent contributions of arousal and stress on attentional failures and successes and how this research may inform understanding of eyewitness reliability.

Eyewitnesses, including those who may also be victims of crimes, are expected to remember relevant and accurate information regarding their witnessed crimes. They are questioned repeatedly and are required to remember small, potentially peripheral, details of crimes, such as the identities of other potential eyewitnesses, and more central details, such as the type of weapons present. These details are often crucial pieces of information used in investigations and in the context of legal proceedings.

It is unlikely that eyewitnesses would be able to accurately recall all details of an event. Further, repeated questioning may leave individuals susceptible to memory errors (Thomas et al., 2010; Chan and LaPaglia, 2011) and inflates confidence in repeated details (Shaw et al., 1996). Researchers have focused a great deal of their work on the impact of retrieval processes, engendered by repeated questioning, on eyewitness memory. Much of this work has demonstrated that retrieval of accurate information when questioning is interleaved with the presentation of new, sometimes incorrect, information. This phenomenon, known as Retrieval Enhanced Suggestibility (RES; cf. Thomas et al., 2010) has its roots in the well-established misinformation effect (for review see, Loftus, 2005). Researchers have found that eyewitnesses are extremely susceptible to questioner demand and are likely to report inaccurate information if asked leading questions (Loftus, 1975; Weinberg et al., 1983; Murphy and Greene, 2016). In addition to demand, researchers have demonstrated that post-event information may disrupt access to original event details (cf. Belli, 1989), or may increase source misattributions (cf. Belli et al., 1994).

Although a large portion of researchers have focused on retrieval of witnessed or experienced events, we present a complementary, but equally important, question to consider: how do attention processes influence the encoding and later retrieval of witnessed or experienced events? Memory distortions and errors are not often investigated as a result of attention and encoding failures. In the laboratory, researchers construct memory experiments such that participants are able to attend to and successfully encode a baseline percentage of information. In a real-life scenario, it cannot be assumed that eyewitnesses encoded 60, 25, or even 1% of the information regarding a crime. We suggest that eyewitness memory and false memory researchers should consider factors that may impact attention, in order to understand the reliability of eyewitness and victim memory.

## The Case for Investigating Attentional Failures and Stress in Eyewitnesses

Humans are rarely able to attend to all visual stimuli in their visual field. Furthermore, they are inundated with distractions, such as their cell phones or their conversational partners. As a result of these realities, they are often susceptible to failures of attention. One such failure of attention is inattentional blindness (Mack and Rock, 1998). This is a failure to notice unusual or unexpected events in a complex visual field. There are many instances in which inattentional blindness can negatively impact individuals' lives. It could be as trivial as a jogger looking at their phone to respond to a text and tripping on a curb to something as serious as a driver looking at a billboard and crashing into a telephone pole. In both of these cases, the individual may have been focusing their attention on one task and failed to notice important information in their environment.

This is directly relevant to witnesses of crimes. It is rare that individuals are ever vigilant for a crime while going about their daily lives. As such, individuals who are questioned as “eyewitnesses” may have been present at a scene but may have not noticed a crime occurring. For example, an individual may be sitting at a bus stop listening to music when two cars crash into each other less than a block away. Since they were looking in the general direction of the crash, a police officer might expect them to be able to provide an eyewitness account of the incident. However, the individual, focusing on their music, may not have been attending to the cars before the crash and only became aware of the incident when sirens alerted them. This would be an example of an eyewitness being expected to provide an account based on their memory for the event, even though they had experienced inattentional blindness. While there is limited research into inattentional blindness for crimes specifically, there is a plethora of research that uses paradigms which could, ostensibly, be applied to eyewitness scenarios.

Additionally, we argue that to understand and predict eyewitness memory accuracy, researchers should not only begin to directly relate attentional failures to later memory distortion, but must also begin to examine components of arousal and stress as these physiological responses to external stimuli may have direct consequences on cognitive processes associated attention and memory formation. The acute stress response can be empirically measured in a lab setting and consists of a biphasic, two-pathway response. The first phase, an experience sometimes colloquially referred to as the “fight or flight” response, provides a sudden burst of energy while the second phase helps repair the body after the stressful experience. Marr et al. (2020) identified a fundamental difference in how different types of memory experts tend to view the impact of stress on memory. Eyewitness memory experts have suggested that stress at encoding impairs eyewitness accuracy, while basic memory experts generally argue that stress at encoding may enhance memory. This discrepancy between groups of researchers has limited the investigation of stress on eyewitness memory. For example, stress researchers often use well-validated stress induction techniques to isolate components of immediate and delayed stress responding and measure physiological changes that result from stress induction. In the past, eyewitness memory researchers have not often used these induction techniques and may have instead inferred a stress response using self-report measures. However more recently researchers have begun to use validated stress induction techniques in eyewitness studies (Krix et al., 2016; Sauerland et al., 2016). In these studies, stress was found to not impair eyewitness encoding. These researchers have even pointed out the discrepancies between the two types of memory research, such as different retention intervals and stress induction techniques, and pointed toward a need for improved methodological rigor within eyewitness research (Sauerland et al., 2016).

Furthermore, to our knowledge, while there are some studies that deal with inattentional blindness under potentially emotionally arousing conditions, there have been no studies which have directly investigated the influence of an acute stress response inattentional blindness. Without this critical information, not only are we failing to come to a consensus regarding the impact of stress on memory encoding, but also failing to even research the impact stress may have on parallel cognitive processes such as inattentional blindness.

Therefore, the current review has two aims. The first is to broadly investigate the basic effect of inattentional blindness in both lab and applied settings. The second aim is to detail the limited research regarding the impact of emotionally arousing stimuli and physiological stress on attention and the impact that stress may have on inattentional blindness. We will conclude by discussing the implications of these emotional and physiological factors on eyewitness memory reliability.

## What is Inattentional Blindness?

The phenomenon now known as inattentional blindness was first demonstrated by Neisser and Becklen (1975), who showed participants three transparent overlapping videos. Two videos depicted people passing basketballs between each other and one depicted a woman with an umbrella walking across the screen. When participants were told to count the number of times one of the teams passed the basketball, 79% of participants missed the umbrella woman walking through the screen. Inattentional blindness, as defined by Mack and Rock (1998), is the phenomenon whereby people tend to miss events that occur in their visual field, no matter how unusual or unexpected those events may be, if their attention is elsewhere. This earlier work by Neisser et al. foreshadows the now famous gorilla experiment (c.f. Simons and Chabris, 1999). In this study, participants counted basketball passes between a group of individuals while a person dressed in a gorilla costume or a woman holding an umbrella walked through the game in clear view of the camera. Participants missed the unusual events 46% of the time.

In a parallel line of research Mack and Rock (1998) had participants look at a small cross on a computer screen and report if the horizontal or vertical arm of the cross was longer. On one of the trials, a small black square appeared in a quadrant of the cross. In this study, an average of 25% of participants failed to notice the black square. However, when participants were simply told to look at the screen without additional attention instructions, all participants noticed the black square. These experiments demonstrated that when people are attending to a task, they can miss other things in their visual field. They also align with Neisser and colleagues' earlier work with more complex and ecologically relevant materials.

While researchers agree that people are susceptible to inattentional blindness, there are conflicting explanations for a potential mechanism for the phenomenon. One possible explanation for inattentional blindness could be that individuals who miss the unexpected stimulus simply did not look at the unexpected stimulus. However, research using eye tracking has shown that this is not the case. There have been several studies that show individuals who were placed in inattentional blindness conditions and individuals who were not given inattentional blindness instructions were equally likely to have eye movements near and even fixate on the stimulus (Koivisto et al., 2004; Beanland and Pammer, 2010).

Another explanation, called inattentional amnesia, was proposed by Wolfe (1999). This explanation purported that the unexpected information is seen and immediately forgotten, rather than not seen at all. Supporting this inattentional amnesia mechanism, Butler and Klein (2009) presented a series of overlapping pictures and words and told participants to report when one of the streams of information (words or pictures) presented the same stimulus back-to-back. They used a recognition task in which participants had lower rates of recognition for the unattended stream of information to show that participants were, in fact, not paying attention to the unattended stream. However, they also found that participants were successfully primed to report information from the unattended stream on a perceptual identification task (completing masked words). The researchers claim that while it is unlikely that participants had explicit awareness of the unattended stream of information, there is evidence that participants were able to perceive the information on some level, and perhaps simply forgot the information at the time of the recognition memory test.

Another study, completed by Ruz et al. (2005), displayed overlapping pictures and letters (which could either spell a word, such as “CLOUD” or a non-word, such as “DLSPR”) to participants. Participants were instructed to attend to either the pictures or the letters and report if stimuli repeated. The researchers used ERPs to find that words were processed differently than non-words by the brain, even when participants were attending to the picture stream of information, not the letter stream. The researchers claimed this may indicate our brains do perceive information, even when that information is not something to which we are attending. Importantly, and contrary to the claims of proponents of inattentional amnesia, this may not be evidence that individuals are *forgetting* information. This is simply because it cannot show that they encoded the information in the first place.

Furthermore, there is ample evidence that runs contrary to an inattentional amnesia mechanism. One such study, completed by Ward and Scholl (2015), asked participants to report on unusual stimuli mid-trial, before there was any chance for perceptual decay. The researchers found that 13% of participants were still unable to report on an unexpected stimulus even when asked immediately following the stimulus on the fourth trial in which something unexpected occurred and had been told to be vigilant for unexpected stimuli. While this rate of IB is fairly low compared to previous studies, this is still significant compared to the control condition in which the stimulus was expected.

These findings align with early work done by Becklen and Cervone (1983), who presented participants with the stimuli created by Neisser and Becklen (1975). However, unlike Neisser and Becklen, they used different versions of the video that ended at various points- at the end, when the woman with the umbrella was in full view, or directly following the woman's exit. They also told another group that the video would end suddenly, at which time the participant would have to immediately report exactly what was presented. The video for this group ended when the woman with the umbrella was on screen. If a tendency to quickly forget encoded information underlies inattentional blindness, not only should the participants for whom the video ended when the woman with the umbrella was on screen have noticed her more than the other participant groups, the participants who were warned ahead of time to describe exactly what was onscreen when the video ended should be able to describe the woman. However, this was not the case. In fact, researchers found that participants who watched the full video or the video that ended once the woman left noticed her 34% of the time, while participants for whom the video ended while she was onscreen noticed her only 7% of the time, regardless of instructions. This study showed no evidence that information was encoded and then forgotten between the video ending and reporting.

An alternative explanation for inattentional blindness was proposed by Mack and Rock (1998). They proposed that inattentional blindness may not necessarily be a failure of seeing, but rather a lack of explicit awareness of the environment. In other words, individuals who experience inattentional blindness may have some sensory awareness of the information in their environment but that sensation may not reach the threshold of awareness necessary to gain full perceptual attention. Essentially, inattentional blindness may be accompanied by unconscious perception (Mack, 2003). This could be what Butler and Klein (2009) and Ruz et al. (2005) found in their studies. There was no evidence that participants were aware of the information and then immediately forgot it. Instead, participants continued to report a lack of awareness even while their brains registered the stimuli.

To this point, research has not been able to pinpoint the specific mechanism that underlies inattentional blindness; however, the proposed mechanisms both predict that people will fail to report unusual events, even when they occur in plain view. This prediction has dramatic applied consequences. As such, a large literature has been devoted to understanding the inattentional blindness phenomenon in more naturalistic and applied settings.

### Inattentional Blindness in Realistic Scenarios

Researchers have examined inattentional blindness in both the lab and in naturalistic settings in order to determine the impact that the phenomenon can have on real-world experiences. For example, Pammer et al. (2015) investigated the potentially dangerous impact that inattentional blindness may have on drivers and bystanders. Participants looked at images of roads (taken from the inside of a vehicle) for 1.5 seconds and were asked to judge whether the image depicted a safe or unsafe environment in which to drive. On a critical trial that contained an unexpected object, participants were also asked if they had seen anything other than the cars, trees, and streetlight. Researchers found that 10% of participants did not report seeing a child running toward the road (the unexpected object). Additionally, over half of participants did not see either an adult or a child standing close by the road. This is an especially concerning depiction of inattentional blindness as the task that participants focused on which made them blind to the road hazards was, quite ironically, looking for road hazards.

Some researchers have also used a lab setting to investigate situations in which eyewitnesses experience inattentional blindness. One such study had participants watch a video of a busy shopping center and either count the number of people wearing a blue shirt, count the number of shopping bags, or just watch the video (Rivardo et al., 2011). Researchers found that 81% of participants who were counting shirts did not notice the theft of a shopping bag, while 62% of people who were counting shopping bags did not notice the theft. Participants who were told to simply watch the video failed to notice the theft only 10% of the time. Importantly, participants who had their attention specifically directed toward stimuli that were directly related to the crime were more likely to notice the crime; however, any task engagement consistently led to higher rates of inattentional blindness than does no task engagement.

Other researchers have looked at rates of inattentional blindness amongst people who are considered “experts” in viewing certain kinds of information. One such study investigated inattentional blindness in radiologists (Drew et al., 2013). Researchers showed a series of lung scans to radiologists and non-radiologists and asked them to identify lung nodules (a common task for radiologists). A gorilla, 48 times the size of the average lung nodule, was on the final scan. Researchers found that 83% of radiologists missed the hidden gorilla while looking for lung nodules, while 100% of non-radiologists missed the hidden gorilla while looking for lung nodules.

Other “experts” have also been found to experience inattentional blindness in their job. For example, Näsholm et al. (2014) found that military personnel tasked with monitoring CCTV footage were susceptible to inattentional blindness for critical information at an alarmingly high rate. Comparing novices and active-duty military personnel, the researchers found that 50% of novices missed a woman placing a suspicious package on the ground and looking into the camera before leaving frame (a task relevant stimulus) and 81% missed a woman in a pirate costume walking into frame and looking at the camera before leaving (a task irrelevant stimulus). Surprisingly, they also found that 61% of the military participants also missed the package stimulus, even though those actions could have severe consequences on a military base, while 76% of military participants missed the pirate. There was no difference in inattentional blindness rates for the participants, regardless of expertise.

Using a more naturalistic approach, Hyman et al. (2009) studied rates of inattentional blindness in college students walking on their campus. They had a person dressed in a clown costume unicycle in a circle near a well-traveled walking path through a campus square and surveyed individuals who walked past the clown. They found that 75% of individuals who were on their cell phones missed the unicycling clown. However, amongst individuals who were not distracted by a cell phone, only 49% of people walking alone missed the unicycling clown, while only 29% of people walking in pairs missed the clown. Common distractions, such as cell phones, led to high rates of inattentional blindness, even for something as absurd, unexpected, and novel as a unicycling clown on a college campus. Interestingly, the individuals walking in pairs noticed the clown more often than the individuals walking alone, a surprising result for those who may think conversation could be a distraction. However, the rate of noticing can be explained by the fact that if one conversational partner noticed the clown, they likely told the other person. Put simply, more observers mean more opportunities for something to be noticed.

In a follow-up study done, Hyman and Wise-Swanson (2014) found that only 6% of 63 individuals who were talking or texting on a cell phone noticed money hanging from a tree branch in the middle of a walking path. Only 19% of the 333 participants who were not engaged in a cell phone saw the money. These data suggest that using a cell phone increases inattentional blindness but that inattentional blindness may still occur even when a cell phone is not in use. The authors suggested that the individuals who were not engaged with their cell phones may have been engaging in some form of mind-wandering. Focusing on their own thoughts may have been an engaging enough task to induce inattentional blindness.

Perhaps more in line with factors that may influence our understanding of eyewitness attentional processes, a naturalistic study completed by Simons and Schlosser (2017) utilized the presence of a gun on the dashboard of a car used in a simulated traffic stop. The simulation was completed by police trainees and experienced police officers as part of a police training exercise. Afterwards, participants were asked if they had noticed the gun plainly displayed in their field of view during the entire traffic stop. Researchers found that the experienced police officers noticed the gun more often than did trainees (67 vs. 42%). However, even amongst experienced law enforcement professionals, who are heavily trained to notice and react to potentially life-threatening objects, a full third of participants missed the unexpected stimulus.

Another study that involved eyewitness attentional processes had participants run behind a researcher while counting the number of times the researcher touched their head (Chabris et al., 2011). At a certain place beside the path were three men in a physical altercation. In this study, only 35% of participants noticed the fight when it took place at night. When it took place in the daylight, 56% of participants noticed the altercation. Noticing rates were also impacted by attentional load. When researchers took away all counting tasks, 72% of participants noticed the fight in the daylight. However, when researchers gave participants two counting tasks, only 42% of participants noticed the fight in the daylight. A full quarter of participants missed something as unusual, violent, and unexpected as a loud physical altercation in broad daylight when simply jogging at a reasonable pace behind another person. This study suggests an important role of attentional load in inattentional blindness, namely that increased attentional load can lead to higher rates of inattentional blindness, especially in a degraded visual field.

### Inattentional Blindness in Emotionally Arousing Scenarios

Inattentional blindness can be experienced by everyone, even when the objects that individuals miss are glaringly obvious to an outside observer or directly relevant to tasks the individuals are trying to complete. It can even be experienced by individuals that may be put in danger by the unexpected and unnoticed object. However, there is a significant factor in many such situations that researchers have yet to investigate. Many of the scenarios that have been discussed in this paper thus far, such as traffic stops (Simons and Schlosser, 2017), CCTV surveillance (Näsholm et al., 2014), or witnessing a crime (Rivardo et al., 2011) are scenarios in which individuals are likely to experience emotional arousal and potentially even a physiological response.

Police officers are taught to be aware of life-threatening danger whenever they are on the job, including during routine traffic stops. Military CCTV operators are charged with ensuring the safety of the base and their fellow soldiers within the base. And, most relevant to the current review, eyewitnesses are often exposed to potentially violent, traumatizing, and/or stressful scenarios of many kinds. Being able to look at the direct effect of stress on inattentional blindness is therefore important in understanding both how inattentional blindness manifests in the context of an acute stress response, and how this interaction may influence the reliability of eyewitness memory.

Unfortunately, to our knowledge, there are no direct experimental manipulations of stress in inattentional blindness studies. However, there are studies that use negative stimuli to investigate how inattentional blindness may be impacted when the unexpected event itself is something potentially emotionally arousing (see Table 1 for a summary of these studies). These stimuli are generally items such as spiders, snakes, or guns. One caveat to these studies is that they do not induce stress prior to exposure to the unexpected stimulus. Rather, it is the unexpected stimuli themselves that are intended to induce a stress response. Although this may better align with a real-world experience of an eyewitness (i.e., it is the crime itself that would likely be the threatening stimulus, not *a prior* scenario), this methodology does not allow for direct examination of acute stress on inattentional blindness. These studies are potentially good indicators of the impact that emotional arousal may have on eyewitness attention but may not provide answers as to how stress impacts eyewitness attention.

**Table 1:** Summary of studies using threatening stimuli in inattentional blindness paradigms.

**Authors**	**Exp**.	**N**	**Experimental paradigm**	**Unexpected (IB) stimulus**	**Noticing rate**		
					**Control stimulus**	**Unexpected stimulus**	**Different from control?**
Simons and Schlosser (2017)		175	Stimulated traffic stop	Gun on dashboard of car	N/A	52.6%	N/A
Näsholm et al. (2014)		171	Watch video and verbally describe events	Person leaving suspicious package or person dressed in pirate costume	*Pirate*: 21%	*Package*: 55%	Yes
Rivardo et al. (2011)		187	Count shirts or bags in a video of a theft of a bag	Individual stealing a bag	*Counting shirts*: 19%	*Counting bags*: 38%	Yes
Beanland et al. (2018)		111	Report specific items from a visual field	Threat word or not threat word	*Non-threat*: 11%	*Threat*: 19%	Yes
New and German (2015)	1	252	Line judgement task	Illustrations of spider and needle	*Needle*: 53%	*Spider*: 81%	Yes
	2	320	Line judgement task	Illustrations of spider, needle, and fly	*Needle*: 53%	*Spider*:80%	Yes
					*Fly*: 73%		No
Gao and Jia (2017)		192	Counting number of color words	Illustrations of threat and non-threat objects	*Low load*: 35.4%	*Low load*: 60.4%	Yes
					*High load*: 19%	*High load*: 35%	No
Wiemer et al. (2013)		120	Line judgement task	Flower picture and spider picture	*Flower*: 58%	*Spider*: 52%	No
Calvillo and Hawkins (2016)	1	168	Searching for a word	Line drawings of threat and non-threat objects	*Non-threat*: 39%	*Threat*: 50%	No
	2	238	Line judgement task	Pictures of threat and non-threat objects	*Non-threat*: 53%	*Threat*: 32%	Yes, threat was noticed less
Stothart et al. (2017)	1	576	Played video game avoiding costly missiles	Square the same color as most costly missiles	*Unrelated color*: 62%	*Most costly*: 30%	Yes, cost was noticed less
	2	595	Played video game avoiding missiles	Square the same color as most costly missiles	*Unrelated color*: 70%	*Most costly*: 53%	Yes, cost was noticed less
	3	599	Played video game avoiding missiles and hitting targets	Square the same color as missiles or target	*Unrelated color*: 78%	*Missiles*: 44% *Target*: 55%	Yes, enemies and friends noticed less
Redlich et al. (2019)	1	277	Line judgement task	Colored square associated with high reward	*No reward associated*: 70.79%	62.11%	No
	2	260	Counting shape bounces	Colored shape associated with high reward	*No reward associated*: 31.03%	29.41%	No

*Note. Not all stimuli from every experiment is included in this table, only those relevant to the present review*.

Regardless, these studies do provide some initial information regarding how inattentional blindness is impacted by threatening stimuli. The first of such studies discussed here was completed by Beanland et al. (2018). The researchers had participants fixate on a cross in the middle of a blank screen, which then flicked briefly to a screen containing four pictures of animals or furniture. The task was to identify the pictures. However, on two of the nine trials the fixation cross was replaced by a threat word (“KILLER”) or a non-threat word (“MERGER” or “MILLER”). Only 22% of participants were able to report one of the pictures, while only 8% were able to report both. Of main interest, however, 19% of participants were able to report the threatening word, while only 11% of participants were able to report the neutral word. This result shows that participants were more likely to report a threatening word than a non-threatening one, even when their attention was on a different task.

There are several more studies that utilize pictures, rather than words, to capture an effect of threat on inattentional blindness. One such study found that 81% of participants who were under conditions that encourage inattentional blindness (a line-length judgment paradigm similar to Mack and Rock, 1998) could detect a line drawing of a spider, compared to only 53% who could do the same for a line drawing of a hypodermic syringe (New and German, 2015). When participants were not under conditions of inattentional blindness, 100% of them were able to detect both the spider and needle. Inattentional blindness was present no matter the stimulus, but may have been reduced by the presence of such a classically negative stimulus as a spider, compared to a relatively newer and less commonly negative stimulus as a needle.

Other researchers have used modern threat objects that are more dangerous than a hypodermic needle. Gao and Jia (2017) found that participants under conditions of inattentional blindness with a low perceptual load were more likely to notice a threat object (e.g. a gun; 60%) than a non-threat object (e.g., a flower; 35%). This aligns with the previous studies, in that participants are more likely to notice threatening objects than non-threatening objects. However, amongst participants who were under a high perceptual load, there was no statistical difference between identifying a threatening (35%) and nonthreatening (19%) object. The low-load task was to report the color words (e.g., blue) from three possible words, while the high-load task was to report the color words from six possible words. Both groups of participants had one second to complete this task.

This is interesting, as it suggests that an even incremental increase in task difficulty could eliminate the effect of threatening objects on inattentional blindness. However, other studies that did not induce perceptual load found a different pattern of results. For example, Wiemer et al. (2013) found that there was no difference in noticing rates of an unexpected picture of a spider (52%) compared to an unexpected picture of a flower (58%). Importantly, these findings contrast those reported by New and German (2015), who used a similar procedure. Furthermore, on a later test of memory, participants were as likely to remember the spider as they were the flower (Wiemer et al., 2013). A reasonable explanation for the difference could be that the flower used by Wiemer et al. (2013) was simply more noticeable than the syringe or fly used by New and German (2015), but it is unclear if this could explain the discrepancies. However, the researchers also found that pictures of spiders resulted in higher skin conductance responses and more saccadic eye movements toward them than did pictures of flowers, even amongst individuals who did not report noticing either picture. This suggests that participants may have processed the stimuli as a threat, even though the threat did not increase rates of noticing the unexpected stimulus.

Further support for the conclusion that threatening stimuli may not impact inattentional blindness comes from a study done by Calvillo and Hawkins (2016), who also used an identification task to assess rates of inattentional blindness. Participants were shown a set of four words around a screen and had one second to find the sport word (e.g., softball). On one trial, an unexpected object appeared in the middle of the screen. The researchers found that there were no differences in noticing rates for threatening (50%) and non-threatening objects (39%). In fact, due to low identification rates of two of their stimuli in particular (a sword and a snake), threatening objects were actually identified less frequently (32%) than were non-threatening objects (53%). In addition, they found that participants were more likely to correctly identify still pictures of animate objects (e.g. a spider or bird; 54%), regardless of potential threat associated with the stimulus, compared to inanimate objects (e.g., a gun or bed; 36%). The authors concluded that it is not the threat that captures attention, but rather if the objects are animate.

In a parallel line of research, studies have shown that penalties and rewards also have little impact on inattentional blindness. In one study, researchers created a computer game in which participants had to avoid enemies, as collisions with enemies would decrease their score, and hit friends, as collisions with friends would increase their score. During this game, an object that matched either the enemy color or the friend color traversed the screen. The researchers found no difference in noticing rates between objects whose colors matched the enemies,' as opposed to friends.' Participants did not notice unexpected objects, even when those objects were associated with a cost in their task (Stothart et al., 2017).

Similarly, when participants were given a task in which certain colors were associated with actual monetary rewards, the reward did not impact inattentional blindness (Redlich et al., 2019). In contrast, although military-trained CCTV operators missed seeing a woman setting down a suspicious package and then leaving in CCTV footage, researchers found that trained and novice operators were more likely to notice the woman setting down a package than they were to notice a woman in a pirate costume staying in frame for an equivalent period of time (Näsholm et al., 2014). This is important for two reasons. First, missing an individual who has a suspicious package on CCTV footage is arguably a more relevant “cost” than missing an object similarly colored to an enemy that makes a participant lose points in a computer game or even a color that is associated with money. Second, being aware of suspicious packages is ingrained in our culture (Morewitz, 2019) and is a well-known potential threat. Because of these reasons, the point that Stothart et al. (2017) made may still stand. In a lab, participants are much less likely to recognize an object as a threat, so extra care must be taken to ensure that participants are reacting to the stimuli in ways the researchers expect. Ecologically valid paradigms, such as those using videos of real people engaging in threatening actions, are potentially the only way we can truly assess how individuals react to threatening stimuli.

The research to date does not suggest a clear picture of how individuals' rates of inattentional blindness would change with the introduction of a threatening stimulus. One thing is clear, however; even in cases in which the threat is clear and present and results in *lower* rates of inattentional blindness, the rates are never reduced to zero. As this relates to eyewitness reliability, the consequences may be errors of omission, memory distortion, and confabulation. Although the highest rate of noticing reached 90% (c.f., New and German, 2015), it is important to note that noticing was defined as simply acknowledging the odd occurrence without including the specifics of the occurrence. Further, this high level of noticing was found in the context of a controlled laboratory experiment and may not represent the conditions experienced by real-world eyewitnesses.

## Arousal, Attention, and Memory

The above studies may be inducing physiological or psychological arousal due to the negative emotions induced by the stimuli. Emotional arousal at the time of encoding has well-studied impacts on later memory. This is relevant to the present review as the physiological and psychological impacts of emotionally arousing stimuli and an acute stressor are similar (Lang and Bradley, 2007; Campbell and Ehlert, 2012). Witnessing or being a victim to a crime may elicit a negative emotional experience. In controlled experiments, researchers have attempted to induce negative emotional arousal to understand the impact it may have on later memory. The literature on emotions and memory is vast; therefore we will focus the present discussion on studies that have examined eyewitness memory specifically or have examined the relationship between negative emotional arousal and attention.

The highly influential Easterbrook hypothesis (1959) proposed that attention narrowing occurs in the context of high emotional arousal. Whereas individuals at moderate levels of arousal are able to attend to many cues in their environment, resulting in a higher level of performance on tasks, individuals with higher levels of arousal may experience attention narrowing, resulting in salience of a subset of cues and obscurity of other cues. For example, in the context of witnessing a crime, negative emotional arousal may result in the salience of a weapon and the hand that is holding the weapon, but indistinctness or ambiguity of non-focal elements (Kocab and Sporer, 2016).

Christianson and Loftus (1991) presented participants with a narrative witnessed event across a series of ordered pictures that contained a target picture wherein a woman either rode the bicycle (neutral event) or was lying on the ground, injured from a bicycle accident (emotional event). The researchers found that participants exposed to the negative picture within the series exhibited better memory for the central detail (color of the woman's coat) but poorer memory for the peripheral detail (color of a car driving in the background) compared to those who viewed the neutral picture.

Yegiyan and Yonelinas (2011) found similar results using individual pictures, rather than a narrative. The pictures were selected from the International Affective Picture System (IAPS; Lang et al., 2008) and depicted scenarios that were designed to elicit varying levels of emotional arousal. Central details were defined as those that would change the description of the event if removed, while peripheral details were defined as those that would not change the description of the event if removed. The authors found that participants who rated the emotional content of negative pictures in the upper half a scale from one (low arousal) to nine (high arousal) exhibited poorer recognition memory for peripheral details than central details in those pictures. The authors concluded that negative emotional arousal likely led to memory narrowing at the highest levels of arousal, a conclusion that aligns with the Easterbrook hypothesis (1959).

Another study compared the memory of police officers for a domestic dispute incident that contained a handgun (the high arousal situation) and a domestic dispute incident that did not contain a handgun (the low arousal situation; Hulse and Memon, 2006). The researchers found that participants who were exposed to the high arousal situation recalled fewer details but were more accurate overall than those exposed to the low arousal situation. While this study did not differentiate between central and peripheral details, this provides further evidence that participants exposed to high levels of emotional arousal are only able to attend to a subset of information while maintaining high levels of performance.

Further, Christianson (1992) suggested that the relationship between negative emotional arousal and memory may depend on whether the emotional stimuli are related to the primary memory task or witnessed event (e.g., if the stimulus that induced emotional arousal was the crime about which the witness is then questioned). Hanoch and Vitouch (2004) considered this idea of arousal-congruent performance and concluded that, in order to see the true impact of emotional arousal on performance, researchers should induce emotional arousal using information that is relevant to the task for which researchers measure performance.

Controlling for attentional capture and fixation, Christianson et al. (1991) found that the emotional arousal-memory effect was unlikely the result of overt attentional processes. As in the earlier study, they exposed participants to a series of sequential pictures that depicted a narrative. The series contained negative and neutral pictures. When participants were restricted to a single fixation on the central object in the critical picture, participants shown the negative pictures had better recall performance for the central detail than did participants who were shown the neutral pictures. As each participant fixated on the same detail for the same length of time, this result cannot be explained by differing levels of overt attention. This same pattern occurred when participants were allowed multiple fixations. Participants who were shown negative pictures had better recall performance for central details and shorter fixation duration than did participants who were shown the neutral pictures.

Kim et al. (2013) recorded the eye movements of participants while exposing them to either negative or neutral picture stories. The authors found that participants had poorer recognition memory for peripheral details in a negative picture story than in a neutral picture story. They also found that participants in general were able to recognize central details after only a short fixation, while peripheral details were better recognized when they were fixated for a longer duration. The impact of fixation length on central details was even less relevant to memory when the pictures were negative, as central details in negative pictures were fixated for significantly less time than were central details in neutral pictures, even though the memory for those details was equal in negative and neutral conditions. However, there was no difference in duration fixation between the negative and neutral conditions for peripheral details, even though memory for those details was poorer in the negative condition than the neutral condition.

Emotional arousal at the time of encoding has an impact on subsequent retrieval. When emotional arousal is induced using negative stimuli, individuals exhibit increased memory performance for details that were central to the event and/or decreased memory performance for details that were peripheral to the event.

## The Biphasic Acute Stress Response

Emotional arousal is only one aspect of the eyewitness experience. Another important factor that none of the previously discussed studies manipulated is the physiological response to an acute stressor. An acute stress response has the potential to occur in both eyewitnesses to and victims of crimes. This response occurs in two phases. The first phase results from the activation of the sympathetic-adreno-medullar (SAM) axis (Godoy et al., 2018). During this phase the body shuts down all unnecessary bodily functions. Adrenaline, a fast-acting and quick-burning source of energy, is released in high volume and heart rate increases.

About 20 min after the stressor has occurred, the body begins to enter the phase two stress response (Kirschbaum et al., 1993). The phase two stress response is directed by the hypothalamic-pituitary-adrenal (HPA) axis (Everly and Lating, 2013). In this phase, the body begins to restart bodily functions and heart rate begins to slow. The production of adrenaline is decreased and replaced with cortisol, a longer-lasting source of energy (Kirschbaum et al., 1993). It is possible to measure participants' response to an acute stressor during this phase by comparing their cortisol levels during a phase two stress response with their cortisol levels prior to stress induction. The peak of this stress response occurs 20 min after the introduction of the stressor (indicated by a peak in cortisol levels around this time; Kirschbaum et al., 1993). The physiological markers of a phase two stress response are generally gone within 24 h of experiencing the stressor.

There are many physiological and psychological similarities between emotional arousal and the acute stress response. Exposure to negative emotionally arousing pictures or videos has been found to increase stress hormones such as adrenaline and noradrenaline (related to the phase one stress response) and cortisol (related to the phase two stress response; Gerra et al., 1996; Codispoti et al., 2003). A well-validated set of emotionally arousing photos, the International Affective Picture System (IAPS; Lang et al., 2008) has been found to reliably induce changes in both overall heart rate and heart rate variability, as well as increase skin conductance (Appelhans and Luecken, 2006; Lang and Bradley, 2007), factors also associated with a stress response (Mackersie and Calderon-Moultrie, 2016; Kim et al., 2018). Therefore, while these responses are discussed separately in the present review, they may have similar impacts on attention and memory and should both be considered when investigating eyewitness reliability.

Although the acute stress response likely accompanies the eyewitness experience, to my knowledge only one study exists that has manipulated the effect of arousal (associated with phase one stress responding) on inattentional blindness. This study had individuals view the gorilla video created by Simons and Chabris (1999) while under varying conditions of physical activity (Hüttermann and Memmert, 2012). They found that participants who did not engage in physical activity noticed the gorilla 20% of the time, while participants under a medium physical load noticed the gorilla 40% of the time. Importantly, high physical load reduced noticing of the gorilla to zero. This pattern of results was replicated in the same paper with a different set of stimuli. However, it is possible that the physical activity itself may have served to divide attention, which had impacts on the cognitive processes independent of those associated with physiological changes.

We propose that researchers should invest efforts in additional studies that examine the acute stress response on attention and inattentional blindness in eyewitness-like scenarios. As opposed to using stimuli that may increase arousal, we argue that new research employing well-established stress induction paradigms should be combined with basic and applied inattentional blindness paradigms. There are many different validated protocols that have been created to induce stress in the lab, such as the Trier Social Stress Test (TSST; Birkett, 2011) or the Socially Evaluated Cold Pressor Test (SECPT; Schwabe and Schächinger, 2018). By incorporating these protocols, this area of research would foster the development of a more comprehensive and generalizable model of eyewitness reliability.

Although little is known about stress and inattentional blindness, there is a small body of work examining stress and attention and a larger body of research focused on stress and memory encoding processes. A meta-analysis by Shields et al. (2016) found that individuals undergoing an acute stress response exhibit improved response inhibition (i.e., ability to withhold responses when necessary; mean effect size, *g*^+^ = 0.296) but impaired cognitive inhibition (i.e., selective attention or ignoring; *g*^+^ = −0.208). Furthermore, this effect is found to hold true regardless of delay between the stressor and the tasks and regardless of cortisol levels, which indicates that the impact of an acute stressor on task performance is the same whether participants were in phase one or phase two of an acute stress response.

One of the studies analyzed by Shields et al. (2016) that demonstrated decreased cognitive inhibition was completed by Sänger et al. (2014). In this study, researchers induced stress in half of participants and then gave them a task during the phase two stress response. This task required participants to report a luminance change in stimuli while ignoring more salient orientation changes in the same stimuli. They found that participants who had been exposed to the stressor made more errors (i.e., missed responding to the task entirely or responded to orientation rather than luminance) than did participants who were not stressed. Furthermore, the electrophysiological data from the same study showed that stressed participants paid less initial attention toward the luminance of the objects (i.e., the task-relevant stimulus) compared to non-stressed participants. This indicates that stressed participants had greater difficulty inhibiting the task-irrelevant information than non-stressed participants.

However, other researchers have found results that do not support the conclusions. Booth and Sharma (2009) exposed participants to a stressor (a loud white noise) during a Stroop task. This task was completed during a phase one stress response. They found that participants who were stressed were better able to ignore irrelevant information than were those who were not stressed. This result aligns with findings presented by Chajut and Algom (2003), who found that participants who experienced a phase one stress response were better able to selectively attend to information than were participants who were not stressed.

In a related line of research, Qi et al. (2018) found that participants in a phase one stress response reported the direction an arrow was pointing faster than non-stressed participants. The authors hypothesized that stressed participants were able to focus only on the important perceptual details (i.e., the head of the arrow) rather than the entire image. All three of these studies took place during a phase one stress response, while the opposite result found by Sänger et al. (2014) occurred during a phase two stress response. This could indicate that a phase one stress response allowed the individuals to engage in more efficient perceptual processing. Rather than impairing cognitive inhibition, a phase one stress response could improve it. This conclusion, however, is muddied by the results of the meta-analysis conducted by Shields et al. (2016), which found stress impaired cognitive inhibition whether the studies tested participants during a phase one or phase two stress response.

Additionally, the impact of stress on individuals' ability to inhibit distractions is unclear. Selective attention could be improved during the phase one stress response (Chajut and Algom, 2003; Booth and Sharma, 2009; Qi et al., 2018). This could mean that participants who experienced a phase one stress response may be better able to focus on a single task and may be less likely to notice an unexpected stimulus. However, Shields et al. (2016) claim that, regardless of stress phase, participants who experienced a stress response may be more susceptible to distraction. Future research needs to specifically investigate the impact of the stress response phase on distractibility in order to make concrete claims in this area.

The impact of stress on attention is not the only factor important to consider in the case of eyewitnesses. Another equally important piece of information is how stress impacts memory. The impact of stress on memory is a well-researched field. In general, participants who experienced a stress response during an emotional event exhibit improved encoding of that event, compared to participants who did not experience a stress response (Cahill et al., 2003; Payne et al., 2007; Henckens et al., 2009). As crimes are often experienced as emotional and may lead to an acute stress response, eyewitness memory may be less susceptible to distortion than previously thought. Furthermore, a stress response at the time of encoding an emotional event has even been found to reduce the negative impact that misleading post-event information has on memory (Hoscheidt et al., 2014). This is another indication that moderate levels of stress may actually be a benefit for eyewitness memory, even downstream.

**Figure 1:**
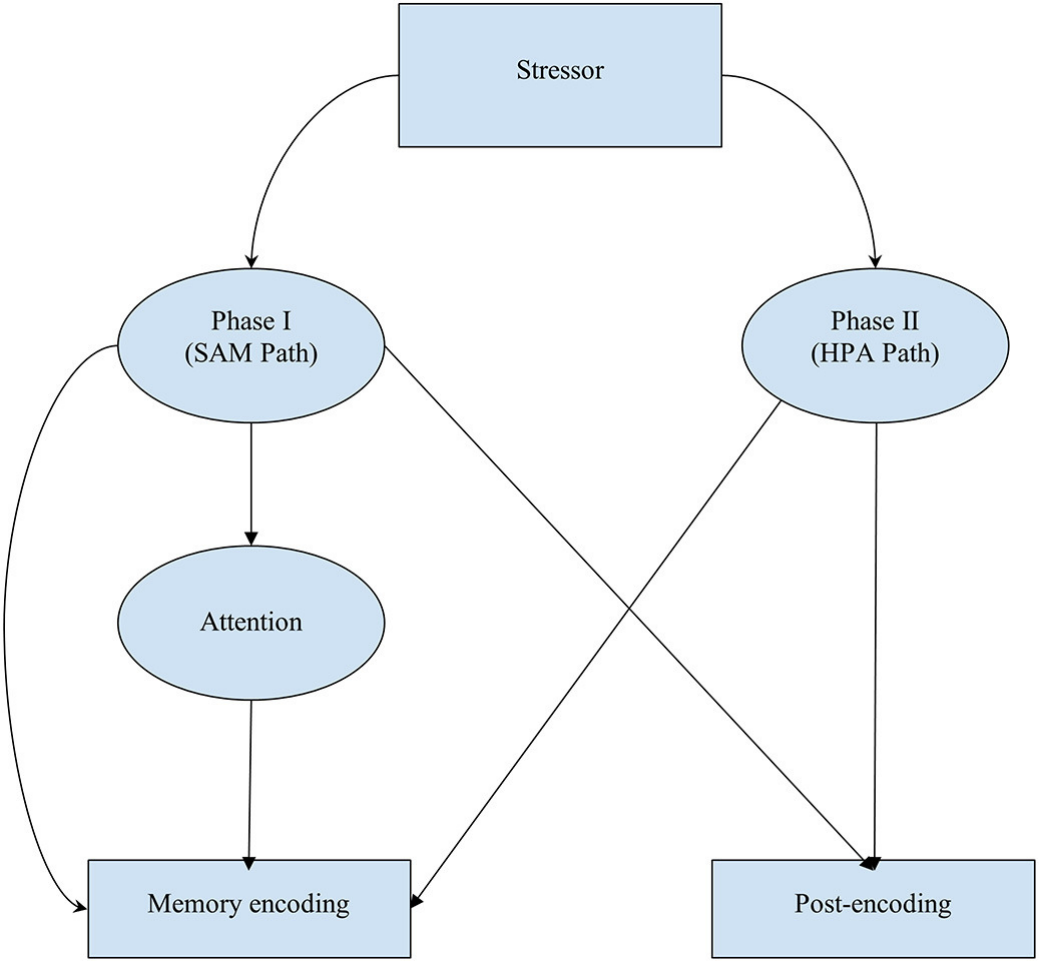
Proposed theoretical model. The stressor results in the simultaneous activation of the SAM pathway and the HPA axis. SAM activation results in the immediate release of adrenaline and norepinephrine, impacting attention, encoding, and post-encoding processes, while the HPA axis is slower acting and results in the release of cortisol, impacting encoding and post-encoding processes. SAM activation may direct attention toward central event details, which subsequently impacts both memory encoding and post-event processes. The release of cortisol may also impact post-encoding processes.

We present a theoretical model based on the present state of the literature that has investigated the interactions between the two phases of the stress response, attention, and memory (Figure 1). A stressor occurs that induces a moderate level of stress, such as witnessing a theft. The stress response occurs in two phases. The phase one stress response, a product of the SAM pathway, pushes the body into a fight-or-flight response (Godoy et al., 2018). The hormones released lead to improved encoding of the event as well as improved post-encoding processes, such as consolidation and post-event retrieval (Gagnon and Wagner, 2016). In addition to encoding processes, the phase one stress response impacts attention. This most likely occurs as a reduction in cognitive inhibition, leading to poorer selective attention and less effective ignoring (Shields et al., 2016). Attention is focused on information that is central to the event, perhaps, in the case of a theft, the perpetrator themselves or the property being stolen. Information that is peripheral to the event, perhaps the identities of other witnesses, is attended to less (Christianson and Loftus, 1991; Yegiyan and Yonelinas, 2011; Kim et al., 2013).

The phase two stress response, occurring around 20 min after the initial stressor, is a product of the HPA pathway (Kirschbaum et al., 1993). At moderate levels, this response has been found to improve both encoding and post-encoding processes (Gagnon and Wagner, 2016). Therefore, a witness to a theft might have improved encoding, consolidation, and post-event retrieval due to the phase two stress response.

Overall, moderate stress would likely have a positive impact on memory for the event providing individuals are attending to details that are central to the event. If individuals are not attending to central details, it is probable that they would not exhibit the beneficial impact that moderate levels of stress can have on memory. It is important for future researchers to begin to incorporate paradigms that encourage individuals to attend to information not central to the target event, such as inattentional blindness paradigms, so we can have a better understanding of how attentional failures may interact with the present model.

Future research into inattentional blindness and stress should begin with a study that investigates the basic impact of a phase one stress response (such as that experienced by eyewitnesses) on inattentional blindness. Participant stress should be induced using a validated stress induction technique, such as the Trier Social Stress Test (TSST) or the Socially Evaluative Cold Pressor Test (SECPT). Immediately following stress induction participants should undergo a basic and well-tested inattentional blindness procedure, such as the line judgement task (Mack and Rock, 1998). The results of this experiment would provide some initial understanding of the impact of experimentally manipulated physiological stress on inattentional blindness. Once this has been determined, future experiments could begin to investigate the downstream consequences stress during attention may have on later retrieval.

## Conclusion

Research into inattentional blindness has been ongoing for decades. There have been a multitude of studies completed in both laboratory and real-world settings that have all come to the same conclusion: humans are susceptible to missing information in our environments, even when that information is seemingly important or unique. This phenomenon may be especially relevant and unfortunate for eyewitnesses, who are already susceptible to memory failures, with this increased susceptibility a function of both attention and encoding failures. Therefore, we present an argument that research should examine the downstream consequences on eyewitness memory failures and distortions as impacted by inattentional blindness. Further, we argue that attention, encoding, and retrieval of witnessed and experienced events should also be investigated within the context of physiological and psychological reactions likely to occur when witnessing or being the survivor of a crime.

The failure to incorporate stress into inattentional blindness research has made it difficult to properly apply inattentional blindness research to eyewitness scenarios. Future research must incorporate experimentally manipulated and valid stress induction into the current inattentional blindness paradigms. Eyewitnesses most commonly experience a phase one stress response during the event, so a phase one stress response would be most applicable to real-world eyewitness scenarios.

Inattentional blindness can be an extremely dangerous attention failure. However, without further research into the direct effect of stress on inattentional blindness, researchers and lawyers alike cannot effectively gauge the efficacy of a potential eyewitness. Researchers must work to combine our knowledge of the impact of stress on attention and inattentional blindness to better serve our scientific understanding of both phenomenon and our ability to impact the legal field.

## Author Contributions

AW drafted the initial manuscript and revised subsequent drafts. AT provided critical feedback and revisions. All authors approved the final version of the manuscript.

## Conflict of Interest

The authors declare that the research was conducted in the absence of any commercial or financial relationships that could be construed as a potential conflict of interest.

## References

[B1] Appelhans B. M. Luecken L. J. (2006). Heart rate variability as an index of regulated emotional responding. Rev. Gen. Psychol. 10, 229–240. 10.1037/1089-2680.10.3.229

[B2] Beanland V. Pammer K. (2010). Looking without seeing or seeing without looking? Eye movements in sustained inattentional blindness. Vis. Res. 50, 977–988. 10.1016/j.visres.2010.02.024 20206648

[B3] Beanland V. Tan C. H. Christensen B. K. (2018). The unexpected killer: effects of stimulus threat and negative affectivity on inattentional blindness. Cogn. Emot. 32, 1374–1381. 10.1080/02699931.2017.1394269 29067866

[B4] Becklen R. Cervone D. (1983). Selective looking and the noticing of unexpected events. Mem. Cogn. 11, 601–608. 10.3758/BF03198284 6669028

[B5] Belli R. F. (1989). Influences of misleading postevent information: misinformation interference and acceptance. J. Exp. Psychol. Gen. 118, 72–85. 10.1037/0096-3445.118.1.72 2522507

[B6] Belli R. F. Lindsay D. S. Gales M. S. McCarthy T. T. (1994). Memory impairment and source misattribution in postevent misinformation experiments with short retention intervals. Mem. Cogn. 22, 40–54. 10.3758/BF03202760 8035684

[B7] Birkett M. A. (2011). The Trier Social Stress Test protocol for inducing psychological stress. J. Visual. Exp. 56:3238. 10.3791/3238 22042290 PMC3227197

[B8] Booth R. Sharma D. (2009). Stress reduces attention to irrelevant information: evidence from the Stroop task. Motiv. Emot. 33, 412–418. 10.1007/s11031-009-9141-5

[B9] Butler B. C. Klein R. (2009). Inattentional blindness for ignored words: comparison of explicit and implicit memory tasks. Conscious. Cogn. 18, 811–819. 10.1016/j.concog.2009.02.009 19328012

[B10] Cahill L. Gorski L. Le K. (2003). Enhanced human memory consolidation with post-learning stress: interaction with the degree of arousal at encoding. Learn. Mem. 10, 270–274. 10.1101/lm.62403 12888545 PMC202317

[B11] Calvillo D. P. Hawkins W. C. (2016). Animate objects are detected more frequently than inanimate objects in inattentional blindness tasks independently of threat. J. Gen. Psychol. 143, 101–115. 10.1080/00221309.2016.1163249 27055078

[B12] Campbell J. Ehlert U. (2012). Acute psychosocial stress: does the emotional stress response correspond with physiological responses? Psychoneuroendocrinology 37, 1111–1134. 10.1016/j.psyneuen.2011.12.010 22260938

[B13] Chabris C. F. Weinberger A. Fontaine M. Simons D. J. (2011). You do not talk about fight club if you do not notice fight club: Inattentional blindness for a simulated real-world assault. I-Perception 2, 150–153. 10.1068/i0436 23145232 PMC3485775

[B14] Chajut E. Algom D. (2003). Selective attention improves under stress: implications for theories of social cognition. J. Personal. Soc. Psychol. 85, 231–248. 10.1037/0022-3514.85.2.231 12916567

[B15] Chan J. C. K. LaPaglia J. A. (2011). The dark side of testing memory: repeated retrieval can enhance eyewitness suggestibility. J. Exp. Psychol. Appl. 17, 418–432. 10.1037/a0025147 21859229

[B16] Christianson S. Å. (1992). Emotional stress and eyewitness memory: a critical review. Psychol. Bull. 112, 284–309. 10.1037/0033-2909.112.2.284 1454896

[B17] Christianson S. Å. Loftus E. F. (1991). Remembering emotional events: the fate of detailed information. Cogn. Emot. 5, 81–108. 10.1080/02699939108411027

[B18] Christianson S. Å. Loftus E. F. Hoffman H. Loftus G. R. (1991). Eye fixations and memory for emotional events. J. Exp. Psychol. 17, 693–701. 10.1037/0278-7393.17.4.693 1832433

[B19] Codispoti M. Gerra G. Montebarocci O. Zaimovic A. Raggi M. A. Baldaro B. (2003). Emotional perception and neuroendocrine changes. Psychophysiology 40, 863–868. 10.1111/1469-8986.00104 14986839

[B20] Drew T. Võ M. L.-H. Wolfe J. M. (2013). The invisible gorilla strikes again: sustained inattentional blindness in expert observers. Psychol. Sci. 24, 1848–1853. 10.1177/0956797613479386 23863753 PMC3964612

[B21] Easterbrook J. A. (1959). The effect of emotion on cue utilization and the organization of behavior. Psychol. Rev. 66, 183–201. 10.1037/h0047707 13658305

[B22] Everly G. S. Lating J. M. (2013). A Clinical Guide to the Treatment of the Human Stress Response. New York, NY: Springer. 10.1007/978-1-4614-5538-7

[B23] Gagnon S. A. Wagner A. D. (2016). Acute stress and episodic memory retrieval: neurobiological mechanisms and behavioral consequences: acute stress and episodic memory retrieval. Ann. NY Acad. Sci. 1369, 55–75. 10.1111/nyas.12996 26799371

[B24] Gao H. Jia Z. (2017). Detection of threats under inattentional blindness and perceptual load. Curr. Psychol. 36, 733–739. 10.1007/s12144-016-9460-0

[B25] Gerra G. Fertomani G. Zaimovic A. Caccavari R. Reali N. Maestri D.. (1996). Neuroendocrine responses to emotional arousal in normal women. Neuropsychobiology 33, 173–181. 10.1159/000119273 8840339

[B26] Godoy L. D. Rossignoli M. T. Delfino-Pereira P. Garcia-Cairasco N. de Lima Umeoka E. H. (2018). A comprehensive overview on stress neurobiology: basic concepts and clinical implications. Front. Behav. Neurosci. 12:127. 10.3389/fnbeh.2018.00127 30034327 PMC6043787

[B27] Hanoch Y. Vitouch O. (2004). When less is more: information, emotional arousal and the ecological reframing of the Yerkes-Dodson Law. Theory Psychol. 14, 427–452. 10.1177/0959354304044918

[B28] Henckens M. J. A. G. Hermans E. J. Pu Z. Joels M. Fernandez G. (2009). Stressed memories: how acute stress affects memory formation in humans. J. Neurosci. 29, 10111–10119. 10.1523/JNEUROSCI.1184-09.2009 19675245 PMC6664979

[B29] Hoscheidt S. M. LaBar K. S. Ryan L. Jacobs W. J. Nadel L. (2014). Encoding negative events under stress: high subjective arousal is related to accurate emotional memory despite misinformation exposure. Neurobiol. Learn. Mem. 112, 237–247. 10.1016/j.nlm.2013.09.008 24055594

[B30] Hulse L. M. Memon A. (2006). Fatal impact? The effects of emotional arousal and weapon presence on police officers' memories for a simulated crime. Legal Criminol. Psychol. 11, 313–325. 10.1348/135532505X58062

[B31] Hüttermann S. Memmert D. (2012). Moderate movement, more vision: effects of physical exercise on inattentional blindness. Perception 41, 963–975. 10.1068/p7294 23362673

[B32] Hyman I. E. Boss S. M. Wise B. M. McKenzie K. E. Caggiano J. M. (2009). Did you see the unicycling clown? Inattentional blindness while walking and talking on a cell phone. Appl. Cogn. Psychol. 24, 597–607. 10.1002/acp.1638

[B33] Hyman I. E. Jr Sarb B. A. Wise-Swanson B. M. (2014). Failure to see money on a tree: in attentional blindness for objects that guided behavior. Front. Psychol. 5:356. 10.3389/fpsyg.2014.00356 24795686 PMC4005951

[B34] Kim H. G. Cheon E. J. Bai D. S. Lee Y. H. Koo B. H. (2018). Stress and heart rate variability: a meta-analysis and review of the literature. Psychiatr. Investig. 15, 235–245. 10.30773/pi.2017.08.17 29486547 PMC5900369

[B35] Kim J. S. C. Vossel G. Gamer M. (2013). Effects of emotional context on memory for details: the role of attention. PLoS ONE 8:e77405. 10.1371/journal.pone.0077405 24116226 PMC3792043

[B36] Kirschbaum C. Pirke K. M. Hellhammer D. H. (1993). The ‘Trier Social Stress Test'—A tool for investigating psychobiological stress responses in a laboratory setting. Neuropsychobiology 28, 76–81. 10.1159/000119004 8255414

[B37] Kocab K. Sporer S. L. (2016). The weapon focus effect for person identifications and descriptions: a meta-analysis, in Advances in Psychology and Law, vol. 1, eds M. Miller and B. Bornstein (New York, NY: Springer), 71–117). 10.1007/978-3-319-29406-3_3

[B38] Koivisto M. Hyönä J. Revonsuo A. (2004). The effects of eye movements, spatial attention, and stimulus features on inattentional blindness. Vis. Res. 44, 3211–3221. 10.1016/j.visres.2004.07.026 15482807

[B39] Krix A. C. Sauerland M. Raymaekers L. H. Memon A. Quaedflieg C. W. Smeets T. (2016). Eyewitness evidence obtained with the Self-Administered Interview© is unaffected by stress. Appl. Cogn. Psychol. 30, 103–112. 10.1002/acp.3173

[B40] Lang P. J. Bradley M. M. (2007). The international affective picture system (IAPS) in the study of emotion and attention, in Handbook of Emotion Elicitation And Assessment, 1st Edn., eds J. A. Coan and J. J. B. Allen (New York, NY: Oxford University Press), 29–46.

[B41] Lang P. J. Bradley M. M. Cuthbert B. N. (2008). International Affective Picture System (IAPS): Affective Ratings of Pictures and Instruction Manual. Technical Report A-8. University of Florida, Gainesville, FL.

[B42] Loftus E. F. (1975). Leading questions and the eyewitness report. Cogn. Psychol. 7, 560–572. 10.1016/0010-0285(75)90023-7

[B43] Loftus E. F. (2005). Planting misinformation in the human mind: a 30-year investigation of the malleability of memory. Learn. Mem. 12, 361–366. 10.1101/lm.94705 16027179

[B44] Mack A. (2003). Inattentional blindness: looking without seeing. Curr. Dir. Psychol. Sci. 12, 180–184. 10.1111/1467-8721.01256

[B45] Mack A. Rock I. (1998). Inattentional Blindness. Cambridge, MA: MIT Press. 10.7551/mitpress/3707.001.0001

[B46] Mackersie C. Calderon-Moultrie N. (2016). Autonomic nervous system reactivity during speech repetition tasks: heart rate variability and skin conductance. Ear Hear. 37(Suppl. 1), 118S−125S. 10.1097/AUD.0000000000000305 27355761

[B47] Marr C. Otgaar H. Sauerland M. Quaedflieg C. W. Hope L. (2020). The effects of stress on eyewitness memory: a survey of memory experts and laypeople. Mem. Cogn. 1–21. 10.3758/s13421-020-01115-4 33237488 PMC8024237

[B48] Morewitz S. J. (2019). Suspicious objects,. in Clinical and Psychological Perspectives on Foul Play, ed S. J. Morewitz (New York, NY: Springer International Publishing), 225–238. 10.1007/978-3-030-26840-4_9

[B49] Murphy G. Greene C. M. (2016). Perceptual load affects eyewitness accuracy and susceptibility to leading questions. Fronti. Psychol. 7:1322. 10.3389/fpsyg.2016.01322 27625628 PMC5003837

[B50] Näsholm E. Rohlfing S. Sauer J. D. (2014). Pirate stealth or inattentional blindness? The effects of target relevance and sustained attention on security monitoring for experienced and naïve operators. PLoS ONE 9:e86157. 10.1371/journal.pone.0086157 24465932 PMC3897661

[B51] Neisser U. Becklen R. (1975). Selective looking: attending to visually specified events. Cogn. Psychol. 7, 480–494. 10.1016/0010-0285(75)90019-5

[B52] New J. J. German T. C. (2015). Spiders at the cocktail party: An ancestral threat that surmounts inattentional blindness. Evol. Hum. Behav. 36, 165–173. 10.1016/j.evolhumbehav.2014.08.004

[B53] Pammer K. Bairnsfather J. Burns J. Hellsing A. (2015). Not all hazards are created equal: the significance of hazards in inattentional blindness for static driving scenes. Appl. Cogn. Psychol. 29, 782–788. 10.1002/acp.3153

[B54] Payne J. D. Jackson E. D. Hoscheidt S. Ryan L. Jacobs W. J. Nadel L. (2007). Stress administered prior to encoding impairs neutral but enhances emotional long-term episodic memories. Learn. Mem. 14, 861–868. 10.1101/lm.743507 18086830 PMC2151024

[B55] Qi M. Gao H. Liu G. (2018). The effect of mild acute psychological stress on attention processing: an ERP study. Exp. Brain Res. 236, 2061–2071. 10.1007/s00221-018-5283-6 29748696

[B56] Redlich D. Schnuerch R. Memmert D. Kreitz C. (2019). Dollars do not determine detection: monetary value associated with unexpected objects does not affect the likelihood of inattentional blindness. Q. J. Exp. Psychol. 72, 2141–2154. 10.1177/1747021819835148 30789089

[B57] Rivardo M. Brown K. Rodgers A. Maurer S. Camaione T. Minjock R.. (2011). Integrating inattentional blindness and eyewitness memory. North Am. J. Psychol. 13, 519–538.

[B58] Ruz M. Worden M. S. Tudela P. McCandliss B. D. (2005). Inattentional amnesia to words in a high attentional load task. J. Cogn. Neurosci. 17, 768–776. 10.1162/0898929053747685 15904543

[B59] Sänger J. Bechtold L. Schoofs D. Blaszkewicz M. Wascher E. (2014). The influence of acute stress on attention mechanisms and its electrophysiological correlates. Front. Behav. Neurosci. 8:353. 10.3389/fnbeh.2014.00353 25346669 PMC4191471

[B60] Sauerland M. Raymaekers L. H. Otgaar H. Memon A. Waltjen T. T. Nivo M.. (2016). Stress, stress-induced cortisol responses, and eyewitness identification performance. Behav. Sci. Law 34, 580–594. 10.1002/bsl.2249 27417874 PMC5129533

[B61] Schwabe L. Schächinger H. (2018). Ten years of research with the Socially Evaluated Cold Pressor Test: data from the past and guidelines for the future. Psychoneuroendocrinology 92, 155–161. 10.1016/j.psyneuen.2018.03.010 29573884

[B62] Shaw I. I. I. J. S McClure K. A. (1996). Repeated postevent questioning can lead to elevated levels of eyewitness confidence. Law Hum. Behav. 20, 629–653. 10.1007/BF01499235

[B63] Shields G. S. Sazma M. A. Yonelinas A. P. (2016). The effects of acute stress on core executive functions: a meta-analysis and comparison with cortisol. Neurosci. Biobehav. Rev. 68, 651–668. 10.1016/j.neubiorev.2016.06.038 27371161 PMC5003767

[B64] Simons D. J. Chabris C. F. (1999). Gorillas in our midst: sustained inattentional blindness for dynamic events. Perception 28, 1059–1074. 10.1068/p281059 10694957

[B65] Simons D. J. Schlosser M. D. (2017). Inattentional blindness for a gun during a simulated police vehicle stop. Cogn. Res. Princip. Implic 2:37. 10.1186/s41235-017-0074-3 28989954 PMC5605606

[B66] Stothart C. R. Wright T. J. Simons D. J. Boot W. R. (2017). The costs (or benefits) associated with attended objects do little to influence inattentional blindness. Acta Psychol. 173, 101–105. 10.1016/j.actpsy.2016.12.012 28039794

[B67] Thomas A. K. Bulevich J. B. Chan J. C. K. (2010). Testing promotes eyewitness accuracy with a warning: implications for retrieval enhanced suggestibility. J. Mem. Lang. 63, 149–157. 10.1016/j.jml.2010.04.004

[B68] Ward E. J. Scholl B. J. (2015). Inattentional blindness reflects limitations on perception, not memory: evidence from repeated failures of awareness. Psychonom. Bull. Rev. 22, 722–727. 10.3758/s13423-014-0745-8 25515671

[B69] Weinberg H. I. Wadsworth J. Baron R. S. (1983). Demand and the impact of leading questions on eyewitness testimony. Mem. Cogn. 11, 101–104. 10.3758/BF03197667 6855553

[B70] Wiemer J. Gerdes A. B. M. Pauli P. (2013). The effects of an unexpected spider stimulus on skin conductance responses and eye movements: an inattentional blindness study. Psychol. Res. 77, 155–166. 10.1007/s00426-011-0407-7 22227916

[B71] Wolfe J. M. (1999). Inattentional amnesia, in Fleeting Memories, ed V. Coltheart (Cambridge, MA: MIT Press), 71–94.

[B72] Yegiyan N. S. Yonelinas A. P. (2011). Encoding details: positive emotion leads to memory broadening. Cogn. Emot. 25, 1255–1262. 10.1080/02699931.2010.540821 21756077

